# Progress in metathesis chemistry

**DOI:** 10.3762/bjoc.15.267

**Published:** 2019-11-15

**Authors:** Karol Grela, Anna Kajetanowicz

**Affiliations:** 1 Faculty of Chemistry, University of Warsaw, Żwirki i Wigury Street 101, 02-089 Warsaw, Poland

**Keywords:** methathesis

Ten years have already passed since the publication of the first thematic issue on olefin metathesis in the *Beilstein Journal of Organic Chemistry* [1], and four years ago, the second part of the thematic issue [2] was published. Now we have the true pleasure to introduce the third one.

Researchers who read these three issues, as well as the followers of the excellent blog "All Things Metathesis" [3] know how much great progress has been made over these years. For example, a number of new highly stereoselective Ru and Mo catalysts have been introduced, solving the problem of *E*- and *Z*-selectivity. Some tagged Ru catalysts can be applied in water and even in biological systems, while Mo and W alkylidenes packed into innovative wax pills are now truly user friendly. Olefin metathesis catalysts can work under homo- or heterogeneous conditions, as well as under continuous flow. The stability of Ru–methylidene species (an attribute important for a successful ethenolysis process) has been significantly improved. Importantly, we have observed a growing number of metathesis examples utilizing very low loading (at the single part-per-million level) of catalysts, which is crucial for the application of this reaction in the production of bulk chemicals. At the same time, much effort was invested in understanding the mechanisms of how new catalysts work and decompose, how macrocycles are formed in ring-closing metathesis, etc. Representative examples of these directions have been the subject of the current, third thematic issue on *Olefin Metathesis*, including highly educative reviews on tandem olefin metathesis–Suzuki–Miyaura cross coupling by Kotha et al. [4], on artificial metalloproteins by Okuda et al. [5], on stereoretentive ruthenium dithiolate catalysts by Mauduit et al. [6], on unsymmetrical NHC ligands by Grisi et al. [7], on polymers by Kudryavtsev [8] and on polyhedral oligomeric silsesquioxanes by Pietraszuk et al. [9]. Finally, Ward and Sabatino wrote a very well-composed review on aqueous olefin metathesis [10]. These tutorials are accompanied by a number of research papers authored by the best experts in the field.

At the same time, the enormous scientific success of this research has – unfortunately – not yet been reflected by a growing number of new industrial openings. No new metathesis-based biorefineries have been built, while the traditional polymer industry seems to prefer ill-definied catalysts, and sadly, no new drugs are being produced by metathesis. On the contrary, Janssen Therapeutics recently announced the discontinuation of the drug Olysio (simeprevir) due to a significant decline in utilization [11]. It is consoling, however, that most of this lack of development is not due to weaknesses of the technology itself. It can rather be attributed to the less-than-favourable business environment and complicated current World's economy. At the same time, a number of recent acquisitions between catalyst producers makes the society fear that a new monopoly may be formed, with the obvious threats for the end-users.

We are therefore looking forward to the future developments in this field. We stay optimistic as we deeply believe that metathesis promoted by modern, innovative catalysts will not be locked-up as a scientific curiosity with little industrial interest. On the contrary, we anticipate that the field will stay competitive and the forthcoming years will bring an explosion of applications utilizing this excellent (and green!) methodology.

It was a great pleasure for us to serve as editors of this thematic issue. We are very thankful to all authors for their first-class contributions. At the same time, we would like to thank the colleagues at the Beilstein-Institut for their professional support and patience.

Karol Grela and Anna Kajetanowicz

Warsaw, October 2019
